# The elimination half-life of crystalloid fluid is shorter in female than in male volunteers: a retrospective population kinetic analysis

**DOI:** 10.1186/s13293-016-0105-7

**Published:** 2016-10-07

**Authors:** Robert G. Hahn

**Affiliations:** 1 Research Unit, Södertälje Hospital, 152 86 Södertälje, Sweden; 2 Karolinska Institutet at Danderyd (KIDS), Stockholm, Sweden

**Keywords:** Sex differences, Crystalloid fluid, Kinetic model

## Abstract

**Background:**

A recent review article suggests that elimination of infused crystalloid fluid might occur faster in females than in males. To study this question, a population kinetic analysis was performed to compare the turnover of buffered Ringer’s solution when infused at different rates in males and females.

**Methods:**

Data were retrieved from seven series of experiments where 44 intravenous infusions of Ringer’s acetate had been given to female volunteers and 67 to male volunteers. Frequent measurements of the blood hemoglobin and the urinary excretion were used as input in a kinetic two-volume model with micro-constants and covariates, using a nonlinear mixed effects software. The key outcome measure was the rate of irreversible elimination of infused fluid, which was expressed as the half-life, obtained as the excreted urine divided by the modeled plasma volume expansion over time.

**Results:**

The half-life amounted to 24 min (95 % confidence interval, 21–27) in the females and 38 min (33–42) in the males. The urinary excretion differed somewhat less than suggested by these figures during the experimental period (3–4 h) because the plasma volume became less expanded in the females. This was due to that fluid that had been distributed peripheral tissues (the interstitium) returned slightly more slowly to the central fluid space (the plasma) in the females. Gender did not serve as a statistically significant covariate to other rate constants in the kinetic model.

**Conclusions:**

The half-life of infused Ringer’s acetate was 60 % longer in healthy male volunteers than in female volunteers.

**Electronic supplementary material:**

The online version of this article (doi:10.1186/s13293-016-0105-7) contains supplementary material, which is available to authorized users.

## Background

Males and females have always been assumed to handle infusion fluids in the same way. However, males have a 5–10 % higher content of body water than females [1], and their urine shows more evidence of fluid retention [2–4]. Pooling of urine-based elimination data in a recent review article suggested that the turnover of crystalloid fluid is faster in females than in males [5]. Therefore, it is possible that the kinetics of crystalloid fluid differs depending in gender. In particular, females could excrete a higher proportion of a fluid load if given the same volume relative to their body weight.

To explore this issue in better detail, the present study estimated the rates of distribution and elimination of infused Ringer’s acetate in both male and female volunteers by a population kinetic analysis that allowed gender to be included as a covariate [6]. Population kinetic analysis of nonlinear mixed effects has gradually become a scientific and industrial standard procedure in drug development [7], and it can be applied to fluid volume kinetics as well [8]. The hypothesis was the elimination but not the distribution of the crystalloid fluid is accelerated in females.

## Methods

This study was based on 111 strictly controlled infusion experiments from 7 studies in which Ringer’s acetate had been administered by intravenous infusion to 70 healthy volunteers with normal serum creatinine. Details on the cohorts are given in Table 1. Results from 6 of the included 7 studies have been published previously [9–14]. The two-stage approach was then used, and a clearance model was utilized in 5 of the publications.

**Table 1:** Demographic data for the cohorts used for population volume kinetic analysis

Study no.	Females/males	Infusions	Age (years)	Body weight (kg)	Fluid volume (ml/kg)	Infusion time (min)	Infusion rate (ml/min/kg)	Study length (min)	Reference no.
1	0/10	10	22 (18–28)	79 (65–101)	20	30	0.666	180	[9]
2	10/0	10	29 (21–39)	63 (58–67)	25	30	0.833	240	[10]
3	0/10	10	32 (24–44)	81 (72–95)	25	30	0.833	240	[11]
4	0/10	19^a^	22 (19–37)	80 (75–100)	5, 10	15	0.333, 0.666	120	[12]
5	0/10	19^a^	31 (28–40)	82 (70–97)	25	15, 30	0.83, 1.66	90	[13]
6	8/0	31^b^	32 (23–46)	61 (52–75)	15, 25	15, 30, 45, 80	0.31, 0.42, 0.55, 0.83, 1.66	135, 150, 165, 310	[14]
7	0/12	12	30 (20–38)	84 (71–98)	25	30	0.833	240	None

Data are the mean (range)

^a^Each male underwent two infusions with different volume or rate. One experiment in one male was excluded due to missed urinary excretion

^b^Six volunteers underwent three to six infusions and two received one infusion each

All subjects gave their approval for participation after being informed about each study’s purpose. The experiments started between 7 and 9 AM in an appropriate facility at Karolinska University Hospital at Huddinge, Söder Hospital in Stockholm, or the University Hospital in Linköping, Sweden. The subjects had been allowed to ingest one glass of fluid and one sandwich before coming to the hospital; exceptions were one study in which the females were in the fasting state [10] and another in which male volunteers ingested 800 ml of water 2 h before the experiment to correct any preexisting dehydration [12].

The volunteers rested for 30 min on a bed to reach a hemodynamic steady state. A cannula was placed in the cubital vein of each arm, one for blood sampling and the other for infusion of Ringer’s acetate. Volunteers were covered in blankets to ensure good thermal comfort. The arm used for blood sampling placed on a body-warm heating pad.

The plasma volume was expanded infusing Ringer’s acetate solution intravenously, using infusion pumps (Flo-Guard 6201, Baxter, Deerfield, IL). The fluid had the following ionic composition (in mmol/l): sodium 130, potassium 4, calcium 2, magnesium 1, chloride 110, and acetate 30 (Baxter Healthcare). During and after these infusions, venous blood (4 ml) was withdrawn to measure the hemoglobin (Hb) concentration and the hematocrit (Hct) on the apparatus used for routine measurements in the hospital’s Clinical Chemistry Laboratory, which was either a Technicon H2 (Bayer, Tarrytown, NY) or a Cell-Dyn Sapphire (Abbot Diagnostics, Abbot Park, IL). The samples were withdrawn in a standardized manner, and duplicate samples collected at baseline ensured a coefficient of variation (CV) of about 1 %. A small discard volume of blood was drawn before each blood collection to preclude an admixture of rinsing solution, and 2 ml of 0.9 % saline was then injected to prevent clotting. Sampling was always performed every 5 min during the infusion and up to at least 30 min afterward, and then, the sampling was spaced at 10–15-min intervals. The baseline sample was drawn in duplicate, and the mean of the two concentrations was used in subsequent calculations. The subjects voided just before the experiments, and this urine was discarded. The volume of the urine excreted during the study was recorded.

### Kinetic analysis

The volume kinetic method used is based on repeated measurement of the blood Hb concentration, which is the inverse of the blood water concentration [6]. Infusion fluids contain almost exclusively water, and Hb changes are therefore an index of the water volume that equilibrates with the circulating blood [7].

The dependent variable in all experiments was simultaneously fitted to two-volume kinetic model with micro-constants after which the influence of covariates was tested sequentially as guided by a reduction of the residual error.

**Fig. 1:**
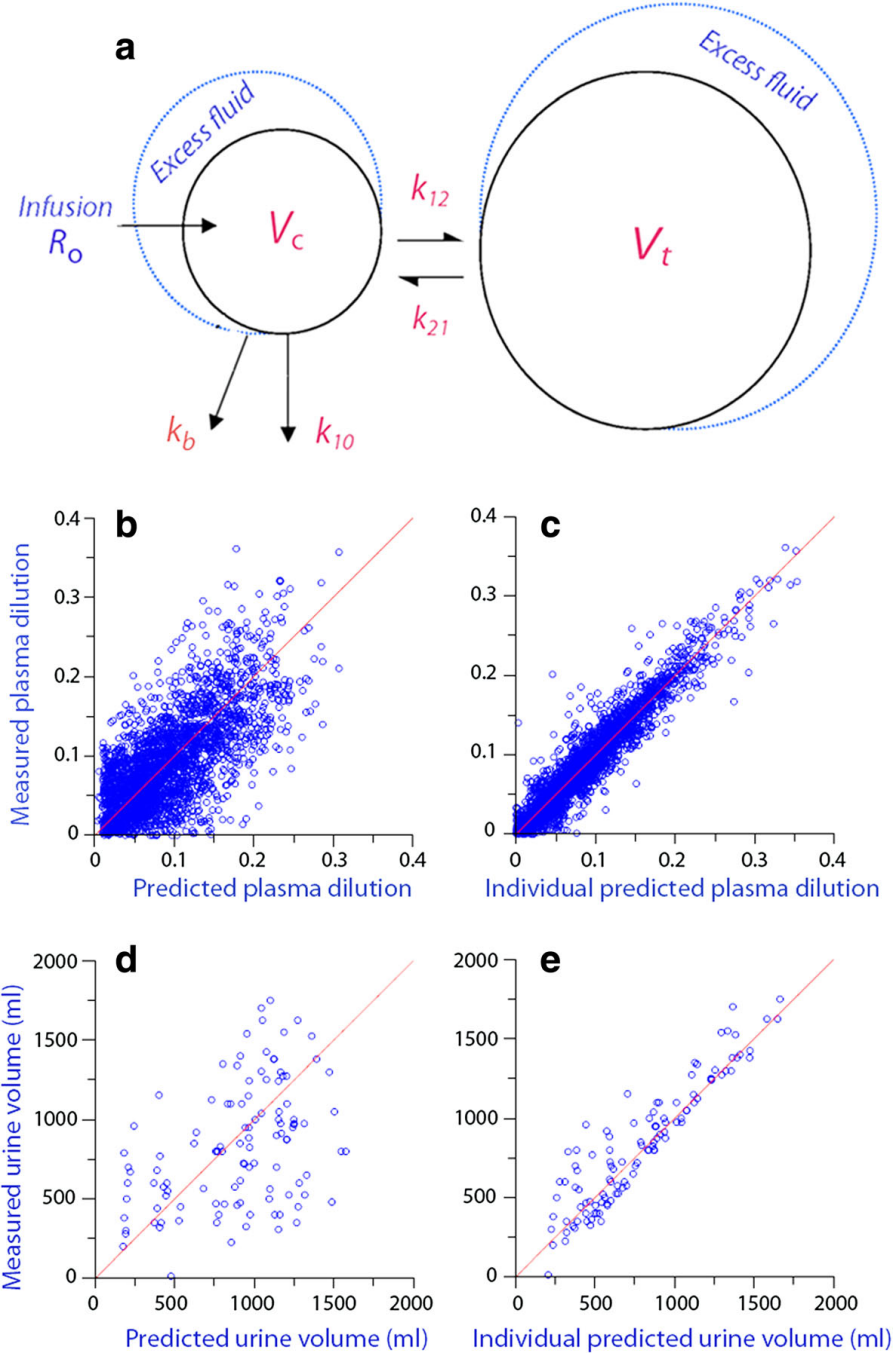
Kinetic model and goodness-of-fit. **a** Schematic drawing of the volume kinetic model. **b** Plasma dilution as predicted by the base model versus the measured plasma dilution for all data points in the 111 experiments. Hence, the predictions are based only on the mean values of *V*_c_, *k*_12_, *k*_21_, *k*_10_, and *k*_b_ for all experiments. **c** Same plot as **b** but after correction of the predicted plasma dilution for covariates, which gives different values to *k*_21_ and *k*_10_ depending on gender. Random distribution around the *solid line* of unity indicates a good goodness-of-fit. **d** Predicted versus measured urinary excretion for all 111 infusions according to the base model. *Each point* represents one patient. **e** Same plot as **d** but after correction for covariates (i.e., gender). Comparing **d** with **e** shows how important gender is when predicting the diuretic response to infused fluid

Fluid was infused at rate *R*_o_ to expand the volume of central body fluid space *V*_c_ to *v*_c_. The fluid distribution to the peripheral body fluid space *V*_t_ was governed by *k*_12_ and its return from *v*_t_ to *v*_c_ by rate constant *k*_21_. The elimination was given by two flows: the urinary excretion was set to the product of the volume expansion of *V*_c_ over time and an elimination rate constant, *k*_10_, and represents irreversible elimination. In contrast, the second flow representing elimination of fluid from the kinetic system is not irreversible as it probably represents filtration of fluid in continuous (non-fenestrated) capillaries that later returned to the plasma via the lymphatic flow [15, 16]. This flow equaled the product of the volume expansion of *V*_c_ and another micro-constant, *k*_b_ (Fig. 1a).

The differential equations for volume changes in the central and peripheral compartments are as follows:

$$ \begin{array}{l}\mathrm{d}{v}_{\mathrm{c}}/\mathrm{d}\mathrm{t}={R}_{\mathrm{o}}-{k}_{10}\left({v}_{\mathrm{c}}-{V}_{\mathrm{c}}\right) - {k}_{\mathrm{b}}\left({v}_{\mathrm{c}}-{V}_{\mathrm{c}}\right) - {k}_{12}\left({v}_{\mathrm{c}}-{V}_{\mathrm{c}}\right) + {k}_{21}\left({v}_{\mathrm{t}}-{V}_{\mathrm{t}}\right)\\ {}\kern2.25em \mathrm{d}{v}_{\mathrm{t}}/\mathrm{d}\mathrm{t} = {k}_{12}\left({v}_{\mathrm{c}}-{V}_{\mathrm{c}}\right) - {k}_{21}\left({v}_{\mathrm{t}}-{V}_{\mathrm{t}}\right)\end{array} $$

The Hb-derived fractional plasma dilution was used to indicate the volume expansion of *V*_c_ resulting from the infusion. The fractional dilution provides a linear relationship between the added fluid volume and the concentration of a marker in the central expandable fluid space (i.e., the plasma) [17]. Hence,

$$ \begin{array}{l}\left({v}_{\mathrm{c}}\hbox{--} {V}_{\mathrm{c}}\right)/{V}_{\mathrm{c}} = \left[\left(\mathrm{H}\mathrm{b}/\mathrm{h}\mathrm{b}\right)-1\Big)\right]\ /\left(1\ \hbox{--}\ \mathrm{b}\mathrm{aseline}\ \mathrm{hematocrit}\right)\\ {}\ \end{array} $$

Symbols in capital letters denote baseline values. A minor correction was made for the effects of blood sampling on the plasma dilution [17].

The primary parameters in the model (*V*_c_, *k*_12_, *k*_21_, *k*_b_, and *k*_10_) were estimated by using the Phoenix software for nonlinear mixed effects (NLME), version 1.3 (Pharsight, St. Louis, MO). The sandwich method was employed as the variance estimator.

The kinetic analysis was performed in two parts. The first part consisted in the development of a *base model*. The lowest residual error was derived with the first-order conditional estimation according to Lindstrom-Bates (FOCE-LB) search routine and the additive error model for the within-subject residual error.

The second part of the kinetic analysis consisted in the addition of covariates. Gender, body weight, and rate of infusion per kilo body weight were sequentially tested as covariates to all five parameters in the model. The threshold for accepting the one curve fit as significantly better (*P* < 0.01) than another curve fit was a reduction of the −2(LL) (log likelihood) by 6.64 points. The base model with the significant covariates added constituted the *final model* and had the lowest residual error of all runs [7].

Previous work shows that the model-predicted irreversible elimination correlates closely with the measured urinary excretion, even when examined over 15-min intervals [10, 14]. Therefore, to increase the stability of the kinetic model, the irreversible elimination half-life (*T*_1/2_) reported here was based on the urinary excretion and derived from the following two equations:

$$ \begin{array}{l}{k}_{10}=\mathrm{urinary}\ \mathrm{excretion}/\mathrm{A}\mathrm{U}\mathrm{C}\ \mathrm{f}\mathrm{o}\mathrm{r}\ \left({v}_{\mathrm{c}}-{V}_{\mathrm{c}}\right)\hfill \\ {}{T}_{1/2}= \ln\ 2/{k}_{10}\hfill \end{array} $$

where AUC is the area under the curve. *T*_1/2_ becomes the same between two groups if the urinary excretion and the AUC for the volume expansion are the same or if relative differences between them cancel out so that their ratio is equal.

Simulations were performed with Matlab R2012b (Math Works Inc., Natick, MA).

Demographic data was reported as the mean (standard deviation), and the kinetic data are reported as the mean (95 % confidence interval). The residual error between the measured values and the computer-simulated (predicted) values were used to describe how well the kinetic model fitted the data. These results were expressed as the median residual error, which shows whether the measured data are systematically higher or lower than the predicted ones (i.e., the bias), and the median absolute residual error, which reflects the degree of deviation of measured from predicted data (i.e., the accuracy).

## Results

Most volunteers were aged 20–30 years and received Ringer’s acetate at rates ranging between 0.33 and 1.66 ml/kg/min (Table 1). The kinetic analysis was based on 2592 data points. The original data are given in Additional file 1.

The covariate search showed that gender affected *V*_c_ and *k*_21_ and that rate of infusion per kilogram body weight also influenced *V*_c_.

The rate constants *k*_12_ and *k*_b_ had no significant covariate.

The estimates of the model parameters derived in the final analysis are shown in Table 2. The three parameters that were affected by covariates had the following form:

**Table 2:** Population kinetic parameters in the final model

	Covariate	Best estimate	2.5 % CI	97.5 % CI	CV%
Kinetic parameter					
tvV_c_ (ml)	–	2521	2185	2858	6.8
tv*k*_12_ (10^−3^ min^−1^)	–	84.0	70.7	97.2	8.0
tv*k*_21_ (10^−3^ min^−1^)	–	54.8	46.3	63.4	8.0
tv*k*_10_ (10^−3^ min^−1^)	–	28.2	24.4	31.6	6.1
tv*k*_b_ (10^−3^ min^−1^)		9.0	7.1	10.9	10.7
Covariate effects					
tv*V*_c_	Dose/min/kg	0.39	0.23	0.55	21.2
tv*V*_c_	Male gender	0.51	0.37	0.64	13.4
tv*k*_21_	Male gender	0.50	0.30	0.71	20.8
tv*k*_10_	Male gender	−0.44	−0.63	−0.27	−19.9

*tv* typical value, *CI* confidence interval, *CV* coefficient of variation

$$ \begin{array}{c}\hfill {V}_{\mathrm{c}} = \mathrm{t}\mathrm{v}{V}_{\mathrm{c}}{\left[\left(\mathrm{volume}/ \min /\mathrm{kg}\right)/\left(\mathrm{volume}/ \min /\mathrm{kg}\right)\ \mathrm{mean}\right]}^{0.39\ }{{\mathrm{e}}^{0.51}}^{\left(\mathrm{female} = 0,\ \mathrm{male} = 1\right)}\hfill \\ {}\hfill {k}_{21} = \mathrm{t}\mathrm{v}{k}_{21}{e^{0.50}}^{\left(\mathrm{female} = 0,\ \mathrm{male} = 1\right)}\hfill \\ {}\hfill {k}_{10} = \mathrm{t}\mathrm{v}{k}_{10\ }{e^{-0.44}}^{\left(\mathrm{female} = 0,\ \mathrm{male} = 1\right)}\ \hfill \end{array} $$

where mean value for the rate of infusion per kilo body weight was 0.82 ml/min/kg and “tv” is the typical value of the parameter. The covariate effect of gender only comes into play for males, as the covariate effect of females is written *e* ^0^ = 1.

The program derived the half-life of the infused fluid volume as a secondary parameter by using the equation *T*_1/2_ = ln 2/*k*_10_. This amounted to 24 min (95 % confidence interval, 21–27) in the females and 38 min (33–42) in the males.

Inclusion of the covariates significantly improved the goodness-of-fit. For the plasma dilution, the mean residual error decreased from −0.007 to −0.001 dilution units and the median absolute residual error from 0.032 to 0.011 dilution units (cf. Fig. 1b, c). The corresponding errors for the urinary excretion were −9, −14, 266, and 82 ml (cf. Fig. 1d, e; all comparisons *P* < 0.001 by Wilcoxon’s matched-paired test).

**Fig. 2:**
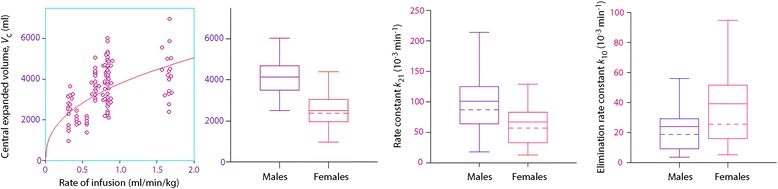
Covariate effects. *V*_c_ and the rate constants *k*_21_ and *k*_10_ obtain different values depending on gender. The mean (*solid line*) and the median (*irregular line*) are displayed within the box, which ends indicate the 25th and 75th percentile limits. The *whiskers* indicate the maximum and minimum values except for extreme outliers, which are not shown (if any)

The results of the covariate analysis are further illustrated in Fig. 2.

The robustness of the model was evaluated by bootstrapping, which result is given in Additional file 2.

**Fig. 3:**
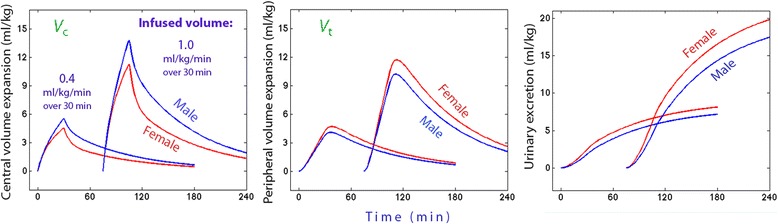
Distribution of infused fluid. Computer simulations of the distribution of fluid between body fluid compartments (*V*_c_, *V*_t_, and urine) when Ringer’s acetate is infused at two different rates in males and females. Fluid distribution is expressed in proportion to the body weight, which averaged 82 kg in the males and 63 kg in the females. The smaller volume expansion of *V*_c_ in the females, which was mostly due to greater accumulation in *V*_t_, limited the short-term impact of the difference in crystalloid fluid half-life on the urinary excretion

Simulations based on the optimal estimates of the kinetic parameters (Table 2) were used to illustrate how two different rates of infusion affected the plasma dilution and the distribution of fluid (Fig. 3).

## Discussion

The present study was based on more than 100 infusion experiments with Ringer’s acetate in healthy volunteers and allowed a comparison between the rates of irreversible elimination of crystalloid fluid depending on gender. The calculations show that half-life of the fluid was almost 60 % longer in the males. This difference would be reflected directly in the urinary excretion if the plasma volume expansion during the experiments had been the same. However, the plasma volume expansion was less pronounced in the females, which somewhat decreased the difference in eliminated fluid volume within the 3-h study period. The reason was that the females retained distributed fluid in peripheral tissues for a longer period of time (Fig. 3).

As a result, not only the plasma/urine ratio but also the plasma/interstitial ratio of infused fluid was lower in the females than in the males. Healthy females then seem to need more fluid per kilo body weight to expand the plasma volume, but slightly less fluid to hydrate the interstitial fluid space, than males do. The brisk urinary excretion still overran the tendency to peripheral edema. Therefore, the present findings agree with the facts that females have less body water [1] and excrete more diluted urine [2–4] than males.

The mechanistic background for the reported gender-associated differences in plasma volume expansion, peripheral volume expansion, and urinary excretion is unclear. The longer persistence might reflect a higher compliance for volume expansion in peripheral tissues in females. One may speculate that the more prompt urinary excretion could be a renal adaptation, governed by a counter-mechanism, aimed to prevent the development of edema in females.

These results were reached by using volume kinetics, which is pharmacokinetics adapted for infusion fluids. The principal modification is that volume kinetics uses the fractional plasma dilution as input function instead of the plasma concentration of a drug [17]. About 50 papers have been published using this approach, but the present study is one of few that uses the strength of population kinetics to arrive at robust results. Each parameter in the model is then allowed to vary according to an individual-specific factor (covariate), which is often gender, age, and body weight [7]. The influence of gender could be evaluated not only on the rate constants governing irreversible and reversible elimination (*k*_10_ and *k*_b_) but also on the volume of distribution (*V*_c_) and the rate constants for distribution (*k*_12_) and redistribution (*k*_21_). Overall, the inclusion of gender as a covariate greatly improved the ability of the kinetic model to predict the input variables, which were plasma dilution and urinary excretion (Fig. 1b–e).

Volume kinetics illustrates how the body handles the infused fluid in a descriptive manner that assumes nonlinear processes. The distribution of infused fluid between body fluid compartments is determined by the four micro-constants, which are inversely proportional to the half-lives of distribution and elimination functions (Fig. 1a). The estimated size of the central body fluid volume at baseline (*V*_c_) is a proportionality factor between plasma dilution and volume expansion and would represent the plasma volume if the Hb molecules were evenly distributed in the blood volume.

The volume of the peripheral space, *V*_t_, can be estimated as *V*_c_ *k*_12_/*k*_21_ and would then average 3.9 l in the females and 6.3 l in the males. These volumes probably reflect mostly the difference in body weight depending in gender. However, they are still smaller than the physiological size of the interstitial fluid space, which can be explained by that volume kinetics only indicates body spaces that can be expanded by fluid [17]. The reason why equilibration of fluid between *V*_c_ and *V*_t_ requires up to 30 min to be completed, as apparent in Fig. 3, is probably that the jelly-like interstitial fluid matrix resists volume expansion [8].

As with pharmacokinetics, the volume kinetic system is not a mechanistic-based model and assumes nonlinear processes that can be described by a summation of linear components. Therefore, the constants may or may not represent identifiable physiological processes. However, an interesting feature of the kinetic model is that some of the fluid that is eliminated from the kinetic model is not recovered as urine. One may speculate that this residual, or reversible, elimination reflects filtration of fluid into parts of the interstitium that do not equilibrate with the plasma. Such filtration occurs through continuous, non-fenestrated capillaries in muscle and connective tissue from where it is slowly returned to the plasma via the lymphatic system [15]. This fluid adds to be edema, although the kinetic analysis suggests that it is eliminated. The ratio *k*_b_/*k*_10_ indicates that one third of the “elimination” occurs by this route. The fluid filtered via *k*_b_ also amounts to one tenth of the fluid that is both distributed and redistributed during the study period (*k*_12_), which takes place via fenestrated capillaries in the kidneys and gastrointestinal tract [16].

The present compilation of data includes 17 experiments, of which 9 were performed in female subjects, where the rate of infusion was extremely high (1.6–1.7 ml/kg/min). A feature of the very highest infusion rates was that the size of *V*_c_ no longer increased with the infusion rate (Fig. 2), which is otherwise the case [7]. A gradual increase in the size of *V*_c_ reflects a progressive reduction of the plasma dilution for higher infusion rates, perhaps reflecting that the acetate in the infused fluid has vasodilatory properties. The increase of *V*_c_ might not only have stopped because the vasodilatory effect of acetate became exhausted but could also be that the mechanisms of elimination operated in an exponential fashion, making a final steady state likely.

The present evaluation shows that the plasma volume expansion and the rate of elimination after infusion of a standardized volume of crystalloid fluid cannot be assumed to be equal in males and females. Gender apparently constitutes a bias in mixed cohorts of volunteers where the kinetics of crystalloid fluid is studied. For good, most of the previously performed studies have included one gender only. Although less desired in the present report, only one gender was included in each of the seven studies included here. However, all experiments were planned and supervised by the author, and during the past 20 years, great care has been taken to perform them in the same highly standardized fashion. The results can therefore be taken to design and power a prospective trial to confirm the observations. The physiological responses to fluid, both with regard to central hemodynamics and water-retaining hormones, should then be patterned and used as time-varying covariates in a fluid volume kinetic analysis of the same type as the present one.

Limitations of the present study include that none of the included series of volunteer experiments were intended to compare gender-dependent differences in fluid volume kinetics. All experiments induced hypervolemia as the volunteers were reasonably normovolemic when the infusions were initiated. No experiments were performed during ongoing menstruation. Notes of the phase of the menstrual cycle were not taken from the females but could, if anything, be expected to prolong the half-life of the crystalloid fluid. Finally, central hemodynamics and other physiological responses to the infusions were not monitored. They could possibly have suggested mechanisms to explain the reported differences in fluid kinetics between females and males.

## Conclusions

A population kinetic analysis based on 111 experiments on 70 volunteers showed that elimination of infused Ringer’s acetate occurs faster in females than in males.

## Additional files

Additional file 1: The Excel file with all original data. (XLS 241 kb)
Additional file 2: Final kinetic output and bootstrap analysis. (DOCX 79 kb)

## Abbreviations

Hb: Hemoglobin
ln: Natural logarithm
*k*_12_ and *k*_21_: Rate constants governing the fluid transfer from *v*_c_ to *v*_t_ and vice versa
*k*_10_: Elimination rate constant determined by urinary excretion
*k*_b_: Rate constant for elimination not explained by urinary excretion
*V*_c_ and *V*_t_: Central and peripheral fluid space expanded by infused fluid to *v*_c_ and *v*_t_

## References

[1] Norberg Å, Sandhagen B, Bratteby L-E, Gabrielsson J, Jones AW, Fan H, Hahn RG (2001). Do ethanol and deuterium oxide distribute into the same water space in healthy volunteers?. Alcohol Clin Exp Res.

[2] Stover EA, Petrie HJ, Passe D, Horswill CA, Murray B, Wildman R (2006). Urine specific gravity in exercisers prior to physical training. Appl Physiol Nutr Metab.

[3] Hahn RG, Waldréus N (2013). An aggregate urine analysis tool to detect acute dehydration. Int J Sport Nutr Exerc Metab.

[4] Johnson P, Waldreus N, Hahn RG, Stenström H, Sjöstrand F (2015). Fluid retention index predicts the 30-day mortality in geriatric care. Scand J Clin Lab Invest.

[5] Hahn RG, Lyons G (2016). The half-life of infusion fluids. Eur J Anaesthesiol.

[6] Owen JS, Fiedler-Kelly J (2014). Introduction to population pharmacokinetic/pharmacodynamic analysis with nonlinear mixed effects models.

[7] Heeremans EH, Proost JH, Eleveld DJ, Absalom AR, Struys MRF (2010). Population pharmacokinetics and pharmacodynamics in anesthesia, intensive care and pain medicine. Curr Opin Anaesthesiol.

[8] Hahn RG, Drobin D, Zdolsek J (2016). Distribution of crystalloid fluid changes with the rate of infusion: a population-based study. Acta Anaesthesiol Scand.

[9] Hahn RG, Bergek C, Gebäck T, Zdolsek J (2013). Interactions between the volume effects of hydroxyethyl starch 130/0.4 and Ringer’s acetate. Crit Care.

[10] Hahn RG, Lindahl C, Drobin D (2011). Volume kinetics of acetated Ringer’s solution during experimental spinal anesthesia. Acta Anaesthesiol Scand.

[11] Drobin D, Hahn RG (2002). Kinetics of isotonic and hypertonic plasma volume expanders. Anesthesiology.

[12] Zdolsek J, Li Y, Hahn RG (2012). Detection of dehydration by using volume kinetics. Anesth Analg.

[13] Svensén C, Drobin D, Olsson J, Hahn RG (1999). Stability of the interstitial matrix after crystalloid fluid loading studied by volume kinetic analysis. Br J Anaesth.

[14] Hahn RG, Drobin D, Ståhle L (1997). Volume kinetics of Ringer’s solution in female volunteers. Br J Anaesth.

[15] Levick JR, Michel CC (2010). Microvascular fluid exchange and the revised Starling principle. Cardiovasc Res.

[16] Woodcock TE, Woodcock TM (2012). Revised Starling equation and the glycocalyx model of transvascular fluid exchange: an improved paradigm for prescribing intravenous fluid therapy. Br J Anaesth.

[17] Hahn RG (2010). Volume kinetics for infusion fluids. Anesthesiology.

