# Supplementary material for: The elimination half-life of crystalloid fluid is shorter in female than in male volunteers: a retrospective population kinetic analysis
Source: Biol Sex Differ. 2016 Oct 7;7:54. doi: 10.1186/s13293-016-0105-7 (PMC5055668; doi:10.1186/s13293-016-0105-7)
Supplement: Additional file 2: — Final kinetic output and bootstrap analysis. (DOCX 79 kb) [file 13293_2016_105_MOESM2_ESM.docx]

**SUPPLEMENTARY MATERIAL 1**

**Hahn: Gender and fluid turnover**


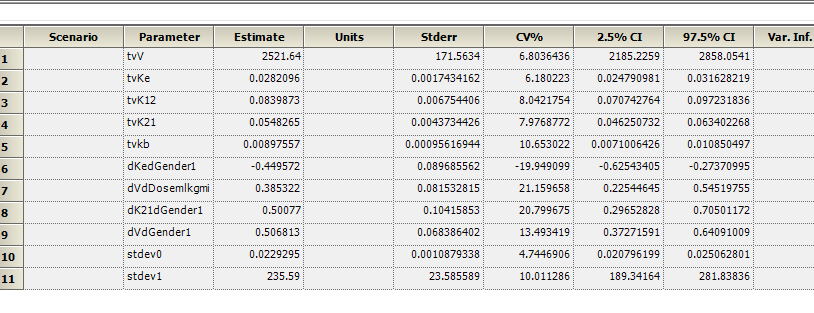


**SUPPLEMENTARY TABLE 1**

Population kinetic parameters in the final model and corresponding parameters derived from bootstrapping (1,000 runs of maximum 99 iterations.).

|  | **Covariate** | **Best estimate** | **2.5% CI** | **97.5% CI** | **CV%** |
| --- | --- | --- | --- | --- | --- |
| POPULATION ANALYSIS |  |  |  |  |  |
| **Kinetic parameter** |  |  |  |  |  |
| tvV_c_ (ml) | - | 2,521 | 2,185 | 2,858 | 6.8 |
| tv*k*_12_ (10^-3^ min^-1^) | - | 84.0 | 70.7 | 97.2 | 8.0 |
| tv*k*_21_ (10^-3^ min^-1^) | - | 54.8 | 46.3 | 63.4 | 8.0 |
| tv*k*_10_ (10^-3^ min^-1^) | - | 28.2 | 24.4 | 31.6 | 6.1 |
| tv*k*_b_ (10^-3^ min^-1^) |  | 9.0 | 7.1 | 10.9 | 10.7 |
|  |  |  |  |  |  |
| **Covariate effects** |  |  |  |  |  |
| tvV_c_ | Dose/min/kg | 0.39 | 0.23 | 0.55 | 21.2 |
| tvV_c_ | Female gender | 0.51 | 0.37 | 0.64 | 13.4 |
| tv*k*_21_ | Female gender | 0.50 | 0.30 | 0.71 | 20.8 |
| tv*k*_10_ | Female gender | -0.44 | -0.63 | -0.27 | -19.9 |
|  |  |  |  |  |  |
| BOOTSTRAP ANALYSIS |  |  |  |  |  |
| **Kinetic parameter** |  |  |  |  |  |
| tvV_c_ (ml) | - | 2,513 | 2,143 | 2,909 | 8.0 |
| tv*k*_12_ (10^-3^ min^-1^) | - | 87.1 | 66.3 | 119.2 | 15.8 |
| tv*k*_21_ (10^-3^ min^-1^) | - | 56.2 | 40.8 | 76.9 | 16.0 |
| tv*k*_10_ (10^-3^ min^-1^) | - | 28.2 | 24.4 | 31.6 | 15.8 |
| tv*k*_b_ (10^-3^ min^-1^) |  | 9.1 | 6.7 | 11.9 | 14.3 |
|  |  |  |  |  |  |
| **Covariate effects** |  |  |  |  |  |
| tvV_c_ | Dose/min/kg | 0.39 | 0.24 | 0.54 | 19.7 |
| tvV_c_ | Female gender | 0.50 | 0.36 | 0.63 | 14.0 |
| tv*k*_21_ | Female gender | 0.50 | 0.16 | 0.87 | 36.4 |
| tv*k*_10_ | Female gender | -0.45 | -0.81 | -0.09 | -41.0 |
|  |  |  |  |  |  |

tv = typical value, CI = confidence interval, CV = coefficient of variation.
